# Structure and Function of the Fecal Microbiota in Diarrheic Neonatal Piglets

**DOI:** 10.3389/fmicb.2017.00502

**Published:** 2017-03-24

**Authors:** Qiaoli Yang, Xiaoyu Huang, Shengguo Zhao, Wenyang Sun, Zunqiang Yan, Pengfei Wang, Shenggui Li, Wangzhou Huang, Shengwei Zhang, Lixia Liu, Shuangbao Gun

**Affiliations:** ^1^College of Animal Science and Technology, Gansu Agricultural UniversityLanzhou, China; ^2^College of Life Science and Engineering, Northwest University for NationalitiesLanzhou, China; ^3^Gansu Research Center for Swine Production Engineering and TechnologyLanzhou, China

**Keywords:** fecal microbiota, diarrhea, functional genes, metagenomics, 16S rRNA gene, neonatal piglets

## Abstract

Diarrhea is a leading cause of increased mortality in neonatal and young piglets. Aberration of the gut microbiota is one important factor in the etiology of piglet diarrhea. However, information regarding the structure and function of the gut microbiome in diarrheic neonatal piglets is limited. To investigate the composition and functional potential of the fecal microbiota in neonatal piglets, we performed 16S rRNA gene sequencing on 20 fecal samples from diarrheic piglets and healthy controls, and metagenomics sequencing on a subset of six samples. We found striking compositional and functional differences in fecal microbiota between diarrheic and healthy piglets. Neonatal piglet diarrhea was associated with increases in the relative abundance of *Prevotella*, *Sutterella*, and *Campylobacter*, as well as *Fusobacteriaceae*. The increased relative abundance of *Prevotella* was correlated with the reduction in *Escherichia coli* and the majority of beneficial bacteria that belonging to the *Firmicutes* phylum (e.g., *Enterococcus*, *Streptococcus*, *Lactobacillus, Clostridium*, and *Blautia*) in diarrheic piglets. The differentially functional gene abundances in diarrheic piglets were an increase in bacterial ribosome, and contributed primarily by the genera *Prevotella*, this indicates a growth advantage of the *Prevotella* in diarrheic conditions. Additional functional gene sets were associated with the reduction of polyamine transport, monosaccharide and sugar-specific PTS transport, amino acid transport, and two-component regulatory system. These profiles likely impact the ability to transport and uptake nutrients, as well as the ability to fight microbial infections in the piglet gut ecosystem. This work identifies a potential role for *Prevotella* in the community-wide microbial aberration and dysfunction that underpins the pathogenesis of piglet diarrhea. Identification of these microbial and functional signatures may provide biomarkers of neonatal piglet diarrhea.

## Introduction

The gastrointestinal microbiota of newborn animals has a profound influence on the host’s health through regulating the intestinal nutritional metabolism, maturation of the immune system, and establishment of the gut barrier (Krajmalnik-Brown et al., 2012; Kabat et al., 2014). Aberration in the structure and function of gut microbiota, termed microbial dysbiosis, is an important factor that impacts several inflammatory intestinal disorders [e.g., acute necrotizing enterocolitis (NEC) and diarrhea], either associated with the presence of specific pathogens (e.g., *Escherichia coli*, *Clostridium perfringens*, *Clostridium difficile*, and *Brachyspira*) (Costa et al., 2014; Minamoto et al., 2014a; Ward et al., 2016; William et al., 2016) or a non-specific dysbiosis (Azcarate-Peril et al., 2011; Pop et al., 2014).

Diarrhea is a leading cause of increased mortality in neonatal and young piglets. Almost half (49%) of piglet deaths are the result of diarrheal infections (Morris et al., 2002). Genetic predisposition and environment factors (i.e., microbial pathogens, nutrition) contribute to the development of this disease. The increased research over the past few decades has advanced our understanding the specific microbial pathogens responsible for triggering piglet diarrhea (Krause et al., 2010; Larsson et al., 2014). Recently, an increased number of neonatal diarrhea cases in the swine industry were found to be associated with additional pathogens (Kongsted et al., 2014; Larsson et al., 2015), making the management of diarrhea even harder. The gut microbiota has been viewed as a contributing factor in the etiology of piglet diarrhea (Hermann-Bank et al., 2015). The gut microbes provide defense against pathogenic invaders by affecting the metabolic state and immune response of the host. When diarrhea develops, the colonization of intestinal pathogenic microorganisms is markedly increased prior to disruption of the intestinal microbial composition and consequently their beneficial functions (Ward et al., 2016). It has been suggested that analyses of the intestinal microbiome may lead to early diagnosis and prevention of intestinal diseases (Li et al., 2012; Raes, 2016). Identification of the gut microbial composition and molecular function that may be involved in the pathogenesis of neonatal piglet diarrhea may provide a new prevention strategy to control this disease.

Pérez-Cobas et al. (2013) suggested that short chain fatty acids producing bacteria from the order *Clostridiales* and corresponding functional genes in aromatic amino acid biosynthesis, tryptophan metabolism, and polyamine biosynthesis were associated with *C. difficile* colonization resistance. In human inflammatory bowel disease (IBD), an imbalance in the abundances of the *Firmicutes* and *Enterobacteriaceae* groups is reported to be corresponded with changes in oxidative stress, carbohydrate metabolism and amino acid biosynthesis (Morgan et al., 2012). Another study exploring the early intestinal microbiome of infants showed that the genes involving in iron acquisition, phosphotransferase systems (PTS), and D-serine metabolism from *E. coli* were associated with NEC (Ward et al., 2016). Together, these findings suggest that the pivotal microbial components form a core of microbial function and the difference of the pivotal microbiota is closely related to different pathophysiologic states.

For suckling piglets, an unstable gut microbial environment and immature immune system make them particularly susceptible to the pathogens, and this increases the risk of diarrhea infection in this stage of life (Bauer et al., 2006). Investigation into the diversity and composition of the gut bacteria in nursing piglets is considerably limited and has mainly focused on longitudinal comparisons on distinct ages or dietary changes around weaning (Thompson et al., 2008; Frese et al., 2015; Slifierz et al., 2015). Frese et al. (2015) studied the fecal microbiome of piglets from birth to weaning and showed that dietary glycans influenced the microbial composition and function. Aberration of the gut microbiota has been identified in neonatal porcine diarrhea and acute necrotizing enterocolitis (Azcarate-Peril et al., 2011; Hermann-Bank et al., 2015). Azcarate-Peril et al. (2011) reported that *Clostridium* spp., *Actinobacteria*, and *Cyanobacteria* were significantly enriched in preterm piglets with NEC. Hermann-Bank et al. (2015) demonstrated that neonatal diarrheic piglets had decreased *Actinobacteria* and *Firmicutes*, as well as increased *Enterococcus* and *E. coli* compared with healthy piglets. However, to the best of our knowledge, the response of functional characteristics to the perturbation of microbiota in piglet diarrhea remains unclear.

In this study, we examined the microbial composition and function of fecal samples from 20 diarrheic and healthy neonatal Large White piglets using 16S rRNA gene sequencing and followed by metagenomic sequencing of a subset of the samples. Our results indicate striking differences in microbial abundance and function between diarrheic and healthy neonatal piglets. Functional genes involved in bacterial ribosome, polyamine transport, monosaccharide and sugar-specific PTS transport, amino acid transport, and two-component regulatory system were associated with neonatal piglet diarrhea.

## Materials and Methods

### Animals and Sample Collection

Piglets were the progeny of six healthy Large White sows that were maintained under identical husbandry practices and epidemic prevention systems in a commercial farm in the Gansu province of China. The farm had no previous history of bacterial and viral infections. Upon delivery, the litters (each litter with 7–11 piglets) were routinely dosed with 2 mg gentamycin sulfate (80,000 IU) to protect against bacterial infection. All piglets were exclusively breast-fed, and were given *ad libitum* access to water. Ear notching was used for individual identification. The general health of each piglet was closely monitored during the first 10 days after birth, and special attention was paid to fecal consistency and disease history. According to these observations, a diarrheic or a healthy piglet was defined based on the criteria described by Hermann-Bank et al. (2015). Briefly, diarrheic piglets were characterized as those suffering from diarrhea for at least two consecutive days with liquid and watery feces that had not received antibiotic therapy prior to sample collection; meanwhile, healthy piglets were those that had never experienced diarrhea and other diseases.

For piglets at seven to 10 days of ages meeting the above conditions, feces were collected using a sterile cotton swab or a slight extrude and immediately frozen on liquid nitrogen. Finally, 20 fecal samples (10 diarrheic and 10 healthy) among those collected samples were further chosen for this study; healthy samples were selected from the litters with diarrheic piglets. All animal experimental protocols were conducted according to the guidelines for the care and use of experimental animals established by the Ministry of Agriculture of China. The project was approved by the Institutional Animal Care and Use Committee (IACUC) of Gansu Research Center for Swine Production Engineering and Technology.

### 16S rRNA Gene Sequencing

Illumina sequencing of 16S rRNA gene was performed to characterize microbial diversity and community composition. Briefly, bacterial genomic DNA was extracted from fecal samples using TIANamp stool DNA kit (TIANGEN, China) according to the manufacturer’s instructions. The quality and concentration of sample was estimated by a NanoDrop 2000 spectrophotometer and agarose gel electrophoresis. The extracted DNA was used as a template for PCR using barcoded primers that flanked the V4 hypervariable region of the bacterial 16S rRNA gene; the primer sequences were 515F (5′-GTGCCAGCMGCCGCGGTAA-3′) and 806R (5′-GGACTACHVGGGTWTCTAAT-3′). Sequencing was performed on an Illumina HiSeq 2500 according to the manufacturer’s instruction for 2 × 250 bp paired-end reads.

The raw sequencing reads were merged using FLASH (v1.2.7), and quality filtering of reads was performed using QIIME pipeline (Caporaso et al., 2010). The acquired sequences were chimera filtered (Haas et al., 2011) using UCHIME algorithm (Edgar et al., 2011) by aligning to the gold database^1^. All 16S rRNA gene sequencing data were deposited in the NCBI short-read archive under BioProject number of PRJNA340296 (Supplementary Table S1).

The high-quality reads were picked into distinct operational taxonomic units (OTUs) using Uparse pipeline (v7.0.1001) with a 97% similarity threshold (Edgar, 2013). They were then taxonomically classified to different levels (phylum, class, order, family, genus, and species) by comparing sequences to the GreenGene database (Desantis et al., 2006) using RDP 3 classifier algorithm (V2.2) (Wang et al., 2007). Species richness (i.e., Observed species), alpha diversity measurement (i.e., Shannon index), and weighted UniFrac distance were calculated using Quantitative Insights Into Microbial Ecology (QIIME) (Werner et al., 2012). The dissimilarity matrices of OTUs were visualized using non-metric multidimensional scaling (NMDS) plots with a conventional cut-off of <0.2 for the stress value.

The statistical differences in alpha and beta diversity of bacterial communities between the two groups were detected using Wilcoxon rank-sum test. The analysis of similarity (ANOSIM) test was used to determine significant difference of microbial communities between diarrheic and healthy groups. Differentially abundant bacterial taxa between the two groups were detect by metastats analysis (White et al., 2009), and only taxa with mean relative abundance > 0.1% in at least one group were considered. *p*-values were corrected for multiple comparisons with Benjamini and Hochberg false-discovery rate correction (*q*-value) (Benjamini and Hochberg, 1995). Significance was considered at *p* < 0.05 and *q* < 0.05.

### Construction of Co-occurrence Networks

Co-occurrence networks can capture the extreme exclusion relationships amongst key microbial taxa. Abundance-based marker genera from 16S rRNA gene sequencing were used to calculate Spearman’s correlation coefficient (*r*) for each group using SPSS 18.0 software (SPSS Inc., 2009). The *p*-value was obtained using an empirical null distribution using 1,000 permutations. Taxa with r above 0.5 or less than – 0.5 were used to construct the correlation networks in Gephi (Jacomy et al., 2009), irrespective of statistical threshold.

### Metagenomic Sequencing

Metagenomic sequencing was performed on a subset of six samples randomly (three diarrheic and three healthy) to investigate the fecal microbial function in neonatal piglets. Sequence libraries were constructed following the manufacturer’s instruction (Illumina, USA). Automated cluster generation was conducted using the Illumina HiSeq 2500 following the 2 × 100 bp paired-end protocol. Raw pair-end reads were first filtered with WindowMasker (Morgulis et al., 2006) to remove low-quality reads (i.e., having > 40% of bases with a quality < Q20 or having > 10 N bases). The host contaminating reads were then removed by aligning clean reads to the swine genome sequences deposited in the NCBI Nucleotide Database (Groenen et al., 2012). On average, 6.29 Gb paired-end reads of high-quality sequences per sample were generated. The metagenomic datasets were deposited to EMBL-ENA’s Sequence Read Archive under the BioProject number PRJEB15296 (Supplementary Table S1).

Clean reads were assembled into contigs for each of the samples using the SOAP *de novo* assembler (v1.06) with the k-mer values of 49, 55, and 59 (Li et al., 2010). The assembly with the largest scaffold N50 was selected. Then the scaffolds were broken at N joints to obtain the scaftigs that did not contain N sequences (Qin et al., 2012). For each sample, the clean data were mapped to scaftigs using SoapAligner (v2.21) with parameters ‘-u, -2, -m 200’ (Li et al., 2009), then the unused reads were binned and repeatedly assembled with the same parameters of the single sample assembly but only one k-mer value, -K 55. Finally, scaftigs with lengths less than 500 bp from single sample and mixed assembly were discarded. A gene catalog was constructed from the assembled scaftigs using MetaGeneMark (Qin et al., 2012). The six samples contained a total of 759,096 ORFs. The gene relative abundance was calculated and renormalized to one according to Qin et al. (2012). Taxonomic assignment of the unigenes was performed using DIAMOND according to the MicroNR database (Version: 2014-10-19) (Buchfink et al., 2015). The relative abundance of a taxon was calculated from the relative abundance of its genes.

### Gene Functional Classification and Ortholog Group Abundance

Protein sequences of the non-redundant genes (432,981) were queried against the Kyoto Encyclopedia of Genes and Genomes (KEGG) database (v.67.0) (Kanehisa et al., 2014) using NCBI blastP (*e*-value ≤ 1e-5). Genes that had alignments with a minimum bit score 60 were assigned into one or more KEGG orthologous (KO). The corresponding tables from gene to KO and from KO to modules were used to build functional modules. The relative abundance of KOs and KEGG modules was calculated by summing the abundance of those genes that annotated to each functional subsystem. We used the ade4 package in R to construct principal component analysis (PCA) based on the abundance of KO profiles.

The comparisons of taxonomic composition and functional profiles (i.e., KOs and KEGG modules) between diarrheic and healthy piglets were performed using metastats with Benjamini and Hochberg correction (Benjamini and Hochberg, 1995; White et al., 2009). Significance was considered at *p* < 0.05 and *q* < 0.1. Linear discriminant analysis (LDA) effect size (LEfSe) analysis was further performed to reveal the significant ranking of abundant modules in diarrheic and healthy samples (Segata et al., 2011). A size-effect threshold of 2.0 on the logarithmic LDA score was used for discriminative functional markers. Spearman’s correlation analysis was used to assess pivotal functional associations with the bacterial taxa, irrespective of statistical threshold.

## Results

### 16S rRNA Gene Profiles in Diarrheic and Healthy Piglets

We sequenced the V4 hypervariable region of 16S rRNA gene to identify the fecal bacterial community diversity and composition in diarrheic and healthy piglets. A total of 1,123,687 high-quality sequences were acquired from 10 diarrheic samples and 10 healthy samples (Supplementary Table S1). The sequences were assigned to 14,386 OTUs based on 97% species similarity; these OTUs mapped to 18 phyla, 30 classes, 53 orders, 77 families and 128 genera.

**FIGURE 1:**
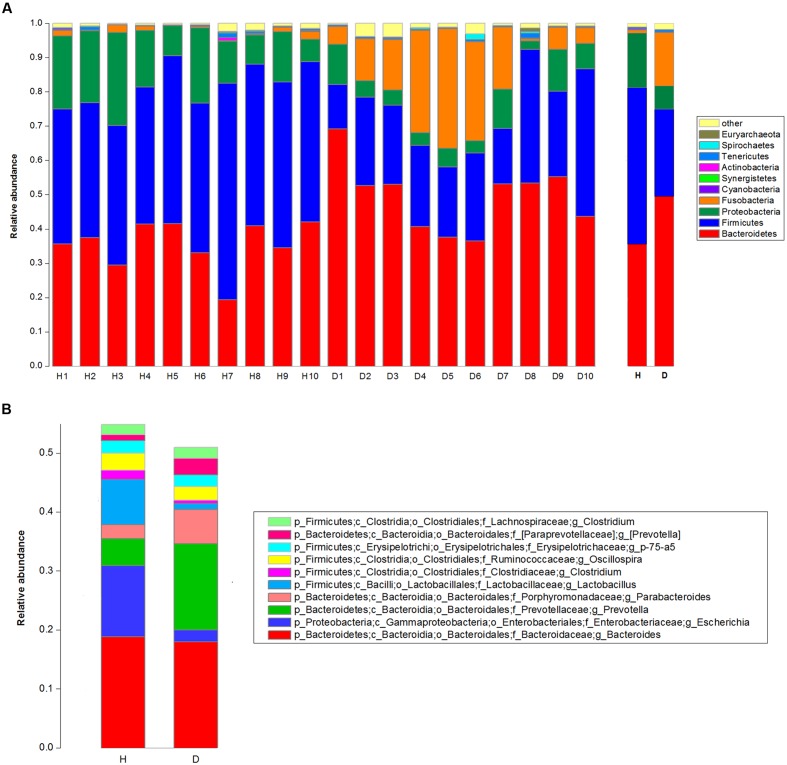
**Taxonomic profiles of the fecal bacteria in diarrheic and healthy piglets from 16S rRNA gene sequencing.** The relative abundance of the top 10 phylum **(A)** and the top 10 genus **(B)** of fecal bacteria present in both diarrheic and healthy piglets. The diarrheic sample and healthy sample is abbreviated as D and H, respectively.

The top 10 phyla and the top 10 genera in relative abundance of the fecal bacteria that present in diarrheic and healthy piglets were displayed in **Figure 1**. *Bacteroidetes* and *Firmicutes* were the most prevalent phyla in both diarrheic and healthy piglets, followed by *Proteobacteria* and *Fusobacteria* (**Figure 1A**). These accounted for 98.04 and 97.23% of the reads for healthy and diarrheic piglets, respectively. Other phyla included *Cyanobacteria*, *Synergistetes*, *Actinobacteria*, *Tenericutes*, *Spirochaetes*, and *Euryarchaeota*; only 0.79 and 1.57% of sequences were unclassified at the phylum level for healthy and diarrheic piglets, respectively. At the genus level, *Bacteroides* was the most dominant in both groups, other major genera included *Escherichia*, *Lactobacillus*, *Prevotella*, *Parabacteroides*, *Clostridium* (member of *Clostridiaceae*), *Oscillospira*, *Phascolarctobacterium*, *p-75-a5*, and *Clostridium* (member of *Lachnospiraceae*); these genera accounted for more than 50% of total sequences (**Figure 1B**).

### Differences of Community Diversity and Bacterial Composition between Diarrheic and Healthy Piglets

**FIGURE 2:**
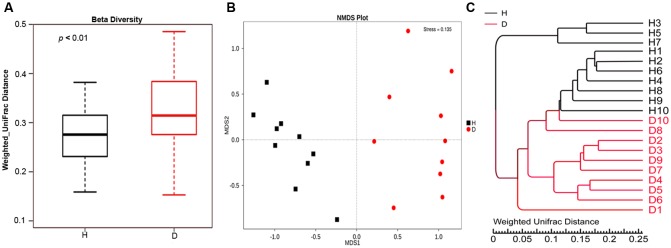
**Comparison of fecal microbial community structure between diarrheic and healthy piglets.** **(A)** Beta diversity (Wilcoxon rank-sum test, *p* < 0.01), **(B)** NMDS plot (ANOSIM *R* = 0.8536, *p* = 0.001) and **(C)** UPGMA tree, all revealing significant differences between diarrheic and healthy piglets based on the weighted UniFrac distances of OTU community. The diarrheic sample and healthy sample is abbreviated as D and H, respectively.

Alpha diversity measures of the fecal bacterial community showed negligible differences between diarrheic and healthy piglets (i.e., Diarrhea vs. Healthy: 6.05 ± 0.16 vs. 6.21 ± 0.20 and 642.30 ± 16.90 vs. 617.20 ± 23.80 for shannon index and species richness, respectively, Wilcoxon rank-sum test, *p* > 0.05), while beta diversity, the variability of OTU community structure between samples, was higher for diarrheic piglets than for healthy controls (Wilcoxon rank-sum test, *p* < 0.01; **Figure 2A**). Similarly, the NMDS plot showing the dissimilarity of microbial community also revealed distinct structure between diarrheic and healthy piglets (**Figure 2B**), and the ANOSIM for differences between the two groups was significant (*R* = 0.8536, *p* = 0.001); this observation was supported by unweighted pair-group method with arithmetic means (UPGMA) analysis based on the weighted UniFrac distances (**Figure 2C**).

**FIGURE 3:**
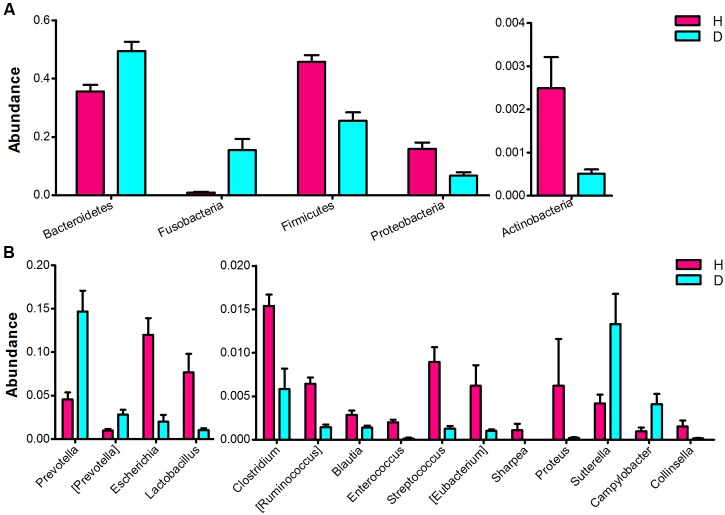
**Comparison of the fecal microbial community of healthy and diarrheic neonatal piglets.** The bacterial phyla **(A)** and genera **(B)** differed between the diarrheic and healthy piglets. The diarrheic and healthy group is abbreviated as D and H, respectively. Bacterial taxa with mean relative abundance greater than 0.1% in at least one group are included. Values are expressed as mean ± SEM. Significance is considered at *p* < 0.05 and *q* < 0.05.

Significant differences in the major taxonomical profiles (mean relative abundance > 0.1%) of fecal microbiota between diarrheic and healthy piglets was further identified using metastats analysis (**Figure 3** and Supplementary Table S2). The relative abundance of the phylum *Bacteroidetes* and the genera *Prevotella* and [*Prevotella*] were significantly elevated in piglets suffering from diarrhea. The relative abundance of the phylum *Fusobacteria* (and the family *Fusobacteriaceae*) in diarrheic piglets was over 4 times (log_2_ fold changes) higher than that in healthy piglets. In contrast, there was a significant decrease in the relative abundance of the phyla *Firmicutes* and *Actinobacteria* in diarrheic piglets. Within the *Firmicutes* phyla, eight genera (*Clostridium*, [*Ruminococcus*], *Blautia*, *Lactobacillus*, *Enterococcus*, *Streptococcus*, *Eubacterium*, and *Sharpea*) exhibited decreased relative abundance. One genus, *Collinsella* (an *Actinobacteria*), had decreased relative abundance in piglets suffering from diarrhea. *Sutterella* and *Campylobacter* (members of the *Proteobacteria* phylum) exhibited increased relative abundances in diarrheic piglets, however, overall there was a significant decrease in the relative abundance of *Proteobacteria* in diarrheic piglets due to the decrease of *Escherichia* and *Proteus*. In addition to the phylum and genus, there were significant differences between the diarrheic and healthy groups in nine classes, eight orders and 13 families (mean relative abundance > 0.1%, *q* < 0.05), see in Supplementary Table S2.

When comparing the taxonomical profiles at the OTU level, 13 OTUs that can be assigned to species showed significant difference in relative abundance between diarrheic and healthy piglets (mean relative abundance > 0.1%, *q* < 0.05; **Table 1**). OTU19 (*Prevotella stercorea*) and OTU25 (*Bacteroides caccae*) were more abundant in diarrheic piglets, whereas OTU1 (*Escherichia coli*), OTU116 (*Enterococcus cecorum*), OTU10 (*Lactobacillus delbrueckii*), OTU79 (*Streptococcus luteciae*), OTU135 (*Collinsella aerofacie*), OTU77 and OTU106 (*Ruminococcus gnavus*), OTU70 (*Eubacterium biforme*), OTU170 (*Sharpea azabuensis*), OTU56 (*Clostridium hathewayi*), and OTU91 (*Clostridium perfringens*) were more abundant in healthy piglets.

**Table 1:** Significant differences in relative abundance of OTUs at species level between diarrheic and healthy piglets.

OTU	Species	Healthy		Diarrhea		*p*-value	*q*-value
					
		Mean abundance	SE	Mean abundance	SE		
OTU19	*Prevotella stercorea*	9.60E-03	3.31E-03	7.18E-02	2.04E-02	9.99E-04	2.01E-02
OTU25	*Bacteroides caccae*	4.30E-04	5.26E-05	1.38E-02	4.49E-03	9.99E-04	2.01E-02
OTU1	*Escherichia coli*	1.20E-01	1.92E-02	2.00E-02	7.83E-03	9.99E-04	2.01E-02
OTU10	*Lactobacillus delbrueckii*	3.28E-02	1.53E-02	9.48E-04	3.55E-04	9.99E-04	2.01E-02
OTU70	*Eubacterium biforme*	4.66E-03	2.25E-03	4.92E-04	9.05E-05	9.99E-04	2.01E-02
OTU56	*Clostridium hathewayi*	5.16E-03	7.56E-04	2.14E-03	5.30E-04	3.00E-03	4.66E-02
OTU77	*Ruminococcus gnavus*	3.62E-03	4.23E-04	5.31E-04	1.86E-04	9.99E-04	2.01E-02
OTU106	*Ruminococcus gnavus*	2.07E-03	6.02E-04	4.72E-04	8.20E-05	9.99E-04	2.01E-02
OTU79	*Streptococcus luteciae*	3.73E-03	1.42E-03	3.98E-04	2.07E-04	9.99E-04	2.01E-02
OTU91	*Clostridium perfringens*	4.05E-03	5.03E-04	2.35E-04	1.14E-04	9.99E-04	2.01E-02
OTU116	*Enterococcus cecorum*	2.00E-03	3.30E-04	1.38E-04	1.01E-04	9.99E-04	2.01E-02
OTU135	*Collinsella aerofacie*	1.51E-03	7.14E-04	1.59E-04	4.70E-05	9.99E-04	2.01E-02
OTU170	*Sharpea azabuensis*	1.09E-03	7.48E-04	9.20E-06	5.09E-06	9.99E-04	2.01E-02


### Microbial Co-occurrence Networks amongst Marker Genera

**FIGURE 4:**
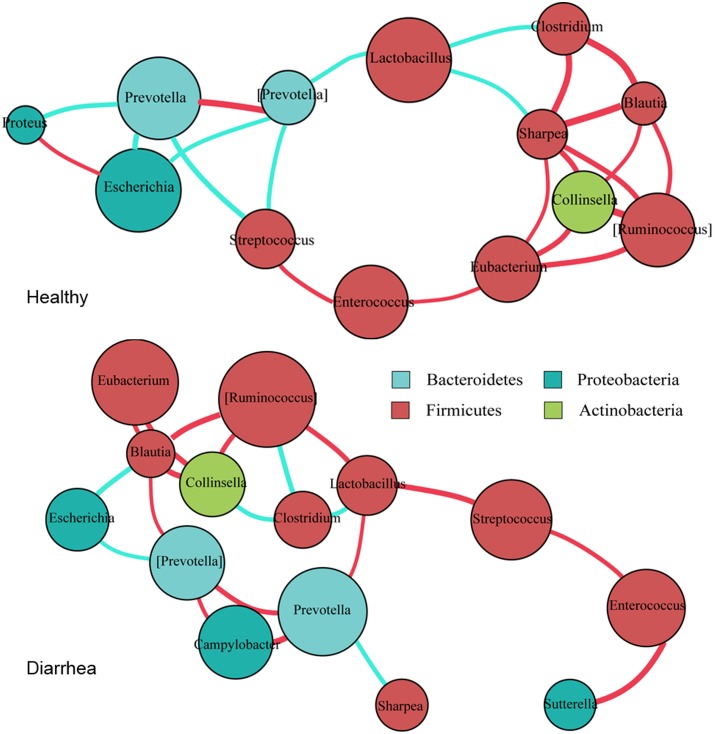
**Co-occurrence networks of bacterial genera constructed on the healthy and diarrheic piglets.** Co-occurrence networks were deduced from 15 marker genera that were identified from 16S rRNA sequencing. Each node represents a genus, the size of each node is proportional to the relative abundance; the color of the nodes indicates their taxonomic assignment. Connection of paired nodes represent Spearman correlation coefficient (red) above 0.5 or below 0.5 (cyan), the thickness of each connection is proportional to the *r*-value.

The co-occurrence networks from the 15 marker genera were constructed for the diarrheic and healthy piglets, based on Spearman’s correlation coefficient (*r*), separately (**Figure 4** and Supplementary Table S3).

In general, there tended to be negative correlations between obligate anaerobes and facultative anaerobes in healthy samples; however, in diarrheic piglets these correlations were either reduced in extent, or became positive. The most obvious example is the negative correlation between the *Prevotella* with the genera *Escherichia*, *Streptococcus*, and *Proteus* in healthy piglets (*r* < -0.6, *p* < 0.05). In diarrhea piglets, the genus *Prevotella* was positively correlated with *Campylobacter* (*r* > 0.6, *p* < 0.01). The protective genus *Lactobacillus* was negatively correlated with most of obligate anaerobes in healthy piglets, but was positively correlated with *Prevotella* and [*Ruminococcus*] in diarrheic piglets (*r* > 0.6, *p* < 0.05). These transitions will probably enable pathogenic microorganisms to thrive under diarrheic conditions. Of a number of obligate anaerobes that were positively correlated with each other in healthy piglets (*r* > 0.6, *p* < 0.05), some of them, such as *Clostridium*, [*Ruminococcus*], *Blautia*, and *Collinsella*, exhibited reduced magnitude or negative correlations in diarrheic piglets (*r* < -0.6, *p* < 0.05). The transition from a positive to negative correlation may indicate competition for oxygen or diversifying selection. Interestingly, *Collinsella* (a member of the phylum *Actinobacteria*) was positively correlated with [*Ruminococcus*], *Blautia* and *Eubacterium* in both groups (*r* > 0.6, *p* < 0.05). This suggests the phylogenetic or functional similarity between the genera. Thus, as the co-occurrence patterns of bacterial communities appear to equate the diversity of intestinal microflora and the health of microenvironment, the gut environment of diarrheic piglets becomes permissive for the development and maintenance of the related taxa in individuals.

### Microbial Functional Characteristics from the Metagenomic Profiles

We utilized metagenome sequencing on a subset samples to determine the microbial gene functions between healthy and diarrheic piglets. Taxonomic profiles of the fecal microbiota in diarrheic and healthy piglets are displayed in **Supplementary Figures S1A,B**. The most prevalent phyla and genera in diarrheic and healthy piglets were similar to that in 16S rRNA gene sequencing, while their relative abundances differed (**Supplementary Figures S1C,D**). We queried the non-redundant genes against the KEGG databases (Kanehisa et al., 2014) using BLAST (Altschul et al., 1990), a total of 4,673 KOs were identified in six piglet fecal samples, which covered 35.27% of the updated gene catalog in the KEGG clustering-based subsystem. The profiles revealed that metabolism for carbohydrate, amino acid, nucleotide, energy and cofactors and vitamins were dominant in both diarrheic and healthy piglets, followed by translation and membrane transport (**Supplementary Figure S2**). Based on the normalized read abundances of KOs, samples were clustered distinctly between diarrheic and healthy piglets by PCA, and 79.53% variation was explained in the first principle component (**Supplementary Figure S3**). At significant threshold of *q* < 0.1, 304 KOs were showed significantly different enrichments in diarrheic and healthy piglets (Supplementary Table S4), and more than half of them were not assigned to the functional pathways/modules or unknown function proteins.

**FIGURE 5:**
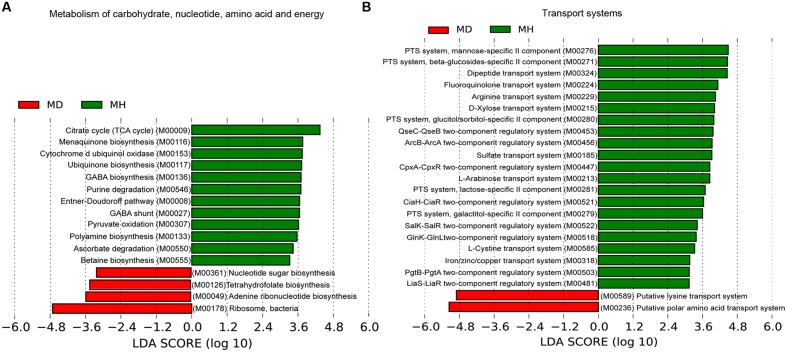
**Association of KEGG modules in the microbial profiles with piglet diarrhea.** Differentially abundant modules between diarrheic and healthy piglets were detected using metastats with Benjamini and Hochberg correction (*p* < 0.05 and *q* < 0.1), and LEfSe analysis was performed to reveal the significant ranking. Modules with a linear discriminant analysis (LDA) score > 2.0 are plotted. Microbial function modules involved in metabolism of carbohydrate, nucleotide, amino acid and energy **(A)**, and transport systems **(B)** are associated with piglet diarrhea.

An investigation of the KOs involved in the functional module levels (i.e., small sets of genes in well-defined metabolic pathways), 397 KEGG modules were detected among all profiles. We compared the modules that covered more than 40% of KOs in the metagenome, there were 52 of these modules were differed in relative abundance between diarrheic and healthy piglets (Supplementary Table S5). LEfSe analysis was performed to reveal the significant ranking of abundant modules (**Figure 5**), and a notable difference was the enrichment of genes involved in ribosome biogenesis; this was accompanied by an increase in the relative abundance of makers for adenine ribonucleotide biosynthesis, tetrahydrofolate biosynthesis, and nucleotide sugar biosynthesis, as well as a decrease in the relative abundance of purine degradation (**Figure 5A**). The enriched level of genes in bacterial ribosome was positively correlated with the high relative abundance of *Prevotella* and *Fusobacterium* (**Table 2**). Furthermore, numerous proteins and enzymes that were involved in ribosomal biogenesis but not annotated to the KEGG modules, such as ribosome recycling factor (K03530), ribonuclease III (K06942), GTP-binding protein (K02428), elongation factor Ts (K07030), adenine deaminase (K07095), and recombination protein RecA (K02078), were also enriched in diarrheic piglets (Supplementary Table S4).

**Table 2:** Distribution of metagenomic reads from pig fecal samples annotated to the ribosome module and Spearman’s correlation between ribosome module and genera.

Module	Genus	Spearman’s correlation	Number of genes	Coverage of the genes (%)
M00178:Ribosome, bacteria	*Prevotella*	0.714	387	12.51
	*Bacteroides*	-0.429	311	10.71
	*Lactobacillus*	-0.714	304	10.46
	*Fusobacterium*	0.6	204	7.02
	*Alistipes*	0.143	202	6.95
	*Clostridium*	-0.257	184	6.33
	*Odoribacter*	0.657	94	3.24
	*Streptococcus*	-0.714	92	3.17
	*Subdoligranulum*	-0.714	84	2.89
	*Holdemanella*	-0.314	83	2.86
	*Phascolarctobacterium*	0.086	75	2.58
	*Oscillibacter*	0.543	71	2.44
	*Sphaerochaeta*	0.486	64	2.20
	*Desulfovibrio*	-0.486	59	2.03
	*Campylobacter*	0.486	58	2.00
	*Parabacteroides*	-0.657	56	1.93
	*Butyricimonas*	0.086	53	1.82
	*Sutterella*	0.486	50	1.72
	*Veillonella*	-0.314	47	1.62
	Other		427	14.70


*A total of 2905 of genes were assigned to the module of ribosome, only genes that can be assigned to genus level were considered. The increase in abundance of bacterial ribosome were contributed by and positively correlated with *Prevotella* and *Fusobacterium*.*

A majority of key metabolism functions exhibited different abundances in diarrheic and healthy piglets (**Figure 5A**). The crucial carbohydrate metabolism modules for pyruvate oxidation, the citrate cycle and the Entner–Doudoroff pathway, were reduced in diarrheic piglets. These were strongly connected to other important functions like energy metabolism. Polyamine, cofactor and vitamin biosynthesis exhibited different relative abundances in diarrheic and healthy piglets (**Figure 5A**). The relative abundance of polyamine and γ-aminobutyric acid (GABA) biosynthesis, cytochrome d ubiquinol oxidase, ubiquinone and menaquinone (vitamin K2) biosynthesis were decreased in diarrheic piglets. These findings support the notion that a decreased microbial protective ability in immune system maturation and inflammation regulation is associated with piglet diarrhea.

The sugar-specific PTS and monosaccharides transport system for carbohydrate uptake were significantly altered in diarrheic piglets (**Figure 5B**). There was a decreased relative abundance of PTS transporter genes for mannose, β-glucoside, glucitol/sorbitol, lactose and galactitol, as well as monosaccharides (i.e., D-xylose and L-arabinose) transport genes in diarrheic piglets. Furthermore, the dipeptide transport system, one mechanism of protein absorption, was also decreased in diarrheic piglets. For the amino acid transport system, the gene relative abundances for fluoroquinolone, arginine, and L-cystine transport decreased; meanwhile, the gene relative abundances for putative lysine transport system and putative polar amino acid transport system increased. Additionally, the gene relative abundances for sulfate transport and iron/zinc/copper transport, which are linked to metal cation metabolism and vitamin B12 synthesis, were significantly decreased in diarrheic piglets. Finally, genes for two-component regulatory systems, type III secretion systems and the twin-arginine translocation system had decreased in relative abundance in diarrheic piglets (**Figure 5B**). The genes for type III secretion systems and QseBC two-component system were correlated with the decrease of *Enterobacteriaceae*; other two-component regulatory systems, such as KdpDE, LiaS-LiaR and GlnK-GlnL were more broadly distributed among the *Firmicutes* (Supplementary Table S6).

## Discussion

Despite well documented evidence implicating gut microbiome in the etiology of diarrhea, the structure and function of the fecal microbiome in diarrheic neonatal piglets remains limited. To gain insight into the compositional and functional characteristics of diarrhea-associated microbiota, we comprehensively evaluate the fecal microbiota on fecal samples of neonatal piglets using 16S rRNA gene and metagenomics sequencing. The striking difference in the bacterial relative abundance and function between diarrheic and healthy piglets supports the notion that alterations in the interactions amongst gut microorganisms are associated with piglet diarrhea. This finding may help better understand the molecular functions of pivotal bacteria on the development of piglet diarrhea.

One major difference in diarrheic piglets was the decreased relative abundance of *Firmicutes* members, including *Lactobacillus*, *Enterococcus*, *Streptococcus*, *Clostridium*, [*Ruminococcus*], and *Blautia* (**Figure 3** and Supplementary Table S2). *Firmicutes* are stable members of the normal gut microbiota in piglets (Slifierz et al., 2015), and several members of this phylum are believed to produce short-chain fatty acids and regulate systemic immune responses (Atarashi et al., 2011; Zhang et al., 2015). Thus, they are likely involved in maintaining energy balance, inhibiting opportunistic pathogens, and protecting the host against excessive intestinal inflammation. For instance, *Lactobacillus* is a commonly used probiotic and is frequently detected in the fecal microbiota of pigs (Li et al., 2012). This bacterium protects against enteric pathogens, and competes with the gram-negatives *Prevotella*, *Sutterella*, and *Campylobacter* for mucosal binding sites (Mann et al., 2014). In this study, *Lactobacillus* and *Streptococcus* were found at lower levels in diarrheic piglets, which are consistent with the previous studies (Costa et al., 2012; Hermann-Bank et al., 2015). These observations indicate that, under diarrheic conditions, the gut environment maintains the competition and abundance of different microbiota; and that the consistent reduction of these clades may thus affect the absorption of nutrients and the ant-inflammatory regulation of the host.

While previous studies have shown that *Enterococcus* increases the risk of neonatal piglet diarrhea (Jonach et al., 2014; Hermann-Bank et al., 2015), the decrease of *Enterococcus* in this work may be due to the specific species of *Enterococcus* encountered. *E. hirae* and *E. duran*s were found to co-occur with pathogenic *E. coli* in the preceding studies (Jonach et al., 2014; Larsson et al., 2014). However, *E. faecalis* and *E. faecium* were the dominant *Enterococcus spp*., as revealed by our metagenomic data (Healthy vs. Diarrhea: 2.95E-04 vs. 3.19E-05 and 1.54E-04 vs. 4.74E-05 for the relative abundance of *E. faecalis* and *E. faecium*, respectively). It has been suggested that *E. faecium* is capable of modulating barrier function of the porcine intestinal mucus (Bednorz et al., 2013; Klingspor et al., 2015). In weaned piglets, the dietary addition of *E. faecalis LAB31* may increase the abundance of *Lactobacillus* and consequently reduce diarrhea (Hu et al., 2015). Thus, we believe that classification at the species level is more appropriate for identifying disease-associated bacteria because the genus level includes species that have varied effects on the host health. Alternatively, a concurrent relationship with other bacteria may also contribute to the decreased relative abundance of *Enterococcus* in this work. In fact, this study indicates that the presence of *Enterococcus*, *Lactobacillus*, and *Streptococcus* were strongly and positively correlated with one other in diarrheic subjects (**Figure 4**).

Increased levels of *Proteobacteria*, including *Sutterella* and *Campylobacter*, were repeatedly reported in intestinal inflammatory disorders (Minamoto et al., 2014b). Although there was an overall decrease in the relative abundance of *Proteobacteria* in diarrheic piglets due to the decrease of *Escherichia* and *Proteus*, the genera *Sutterella* and *Campylobacter* were observed with significantly increased relative abundance in diarrheic piglets, suggesting a pathogenic role in the development of piglet diarrhea. The increases of *Sutterella* and *Campylobacter* may be caused by decreased competition from *Lactobacillus* (Mann et al., 2014). *E. coli* is a major opportunistic pathogen that causes infection in NEC and diarrhea (Hermann-Bank et al., 2015; Ward et al., 2016). However, this bacterium was predominant and negatively correlated with *Prevotella* in healthy piglets (**Figures 2**, **4**). It appears that *E. coli* in this study is non-pathogenic.

*Prevotella* is increasingly gaining attention not only because of its ability to degrade plant polysaccharides (Ivarsson et al., 2014), but also its proinflammatory property (Scher et al., 2013). This genus is present in nursing pigs with very low abundance and increases rapidly in weaned pigs when a plant-based diet is introduced (Alain et al., 2014; Frese et al., 2015). Intriguingly, we found that *Prevotella* was more dominant in diarrheic neonatal piglets. This is in line with the study that the *Prevotella-*dominated microbiome in inflammatory condition is a community shift away from *Bacteroides* and a large amount of beneficial microbes such as Group XIV *Clostridia*, *Blautia*, and *Lachnospiraceae* clades (Scher et al., 2013). Besides, several studies suggested a negative correlation between *Prevotella* and *Escherichia.* Kang et al. (2013) reported that the *Prevotella* cluster displayed a negatively correlated pattern with the *Enterobacteriaceae* cluster (e.g., *Salmonella*, *Escherichia*/*Shigella*, and *Citrobacte*) in healthy children. In contrast, Pop et al. (2014) revealed a negative correlation between *Prevotella* and *Escherichia* in young children with diarrhea. De et al. (2010) suggested that suppression of pathogenic *Escherichia*/*Shigella* by *Prevotella* might result in a lower incidence of gastrointestinal disorders in African children. We observed a strong negative correlation between *Prevotella* and several facultative anaerobic genera (e.g., *Escherichia*, *Proteus*, and *Streptococcus*) in healthy piglets; however, this correlation was disturbed in diarrheic condition (**Figure 4** and Supplementary Table S3). These observations suggest that the community-wide interrelationship of gut microbiota is altered in diseased subjects. The alteration in this study may confer a higher risk for trigger diarrhea and explain the changes in relative abundance of these microbes in diarrheic piglets.

*Fusobacterium* is considered to be an inflammatory microorganism and a prognostic biomarker that inhibits T-cell responses and promotes the expression of inflammatory factors (Nosho et al., 2016; Wei et al., 2016). The increased relative abundance of *Fusobacteriaceae* in diarrheic piglets has also been reported in neonatal porcine diarrhea (Hermann-Bank et al., 2015) and horses with colitis (Costa et al., 2012). This suggests that *Fusobacterium* may be a contributing factor in the etiology of piglet diarrhea.

Beyond microbial composition, the microbial functional profiles in diarrheic piglets also differed from those in healthy piglets. The fecal microbiome of diarrheic piglets enriched ribosome biogenesis genes, which were accompanied by increase in adenine ribonucleotide and nucleotide sugar biosynthesis, as well as decrease in purine degradation (**Figure 5A**). It is generally known that ribosome biogenesis is essential for bacterial cell viability. In this work, we observed that genes involved in bacterial ribosome were contributed primarily by *Prevotella* and *Fusobacterium* (**Table 2**). This confirms the significantly increased relative abundance of *Prevotella* and *Fusobacterium*. A recent study on gene function prediction in *E. coli* has revealed new functions involving in cell adhesion, ribosomal protein biogenesis, and degradation (Vlasblom et al., 2015). Interactions between membrane protein genes and ribosome-dependent ATPase genes in *E. coli* have also been linked to oxidative phosphorylation and protein synthesis (Babu et al., 2011). One hypothesis is that ribosome biogenesis genes in *Prevotella* has similar functions to *E. coli*, and that these genes may be as an indicator of neonatal piglet diarrhea. Besides, ribosome biogenesis requires enormous amount of cellular energy, and thus may be tightly coupled with the process of oxidative phosphorylation.

Metabolic functions may also play a role in the development of piglet diarrhea. There was a decreased abundance of functions for ubiquinone and menaquinone (vitamin K2) biosynthesis, polyamine biosynthesis, GABA biosynthesis and GABA shunt. Ubiquinone (coenzyme Q) is vital for cellular energy production, but also an electron carrier in the membrane-bound electron transport chain that protects cells against damage (Søballe and Poole, 2000). Polyamine has been shown to have anti-inflammatory effects by the suppressing production of proinflammatory cytokines IL-12 and IFN-γ (Haskó et al., 2000), the increase of polyamine biosynthesis was linked to the colonization resistance of *C. difficile* (Pérez-Cobas et al., 2013). Alternatively, microbial processing of putrescine can produce GABA which is thought to stimulate the release of glucagon-like peptide1 from intestinal cells to maintain energy homeostasis (Gameiro et al., 2005). Thus, these metabolites suggest a disturbed energy metabolism or a decreased protection against inflammatory and oxidative (or acid) stress in diarrheic piglets. It has been suggested that polyamine and GABA biosynthesis possibly correlated with the decrease of *Enterobacteriaceae* (Søballe and Poole, 2000; Wunderlichová et al., 2014). Also, several energy metabolism pathways seem important, such as pyruvate oxidation, the citrate cycle, and the Entner–Doudoroff pathway, suggests that the potential of microbes to assimilate essential compounds and all possible energy substrates is decreased when piglets are diarrheic.

Our data also shows broad decreases in various transport systems (e.g., amino acid transport, sugar-specific PTS transport, monosaccharides transport) in diarrheic piglets (**Figure 5B**). *E. coli* utilization of L-ascorbate (i.e., vitamin C) under anaerobic condition is mediated by PTS-type transporters (Zhang et al., 2003). In the absence of glucose, L-arabinose activates the operon’s controlling gene in *E. coli* that drive arabinose-catabolization as a source of carbon and energy (Schleif, 2010). A separate metatranscriptome analysis supported this idea, as high expression of PTS genes belonging to *Streptococci* for rapid uptake and fermentation of available carbohydrates was observed (Zoetendal et al., 2012). The microbial genes for the degradation of sugar substrates from breast milk are the major source of nutrition for neonatal piglets. The decrease in these transport genes hampers phosphorylation of sugar substrates and suggests an imbalanced energy metabolism. Taken together, these observations strongly suggest that consistent reductions of transport genes have profound effects on the energetic metabolism and possibly play a role in the development of piglet diarrhea.

Defects in the type III secretion system render a bacterium non-pathogenic (Gong et al., 2010); the QseBC two-component system was associated with bacterial quorum sensing and virulence factors expression (Weigel and Demuth, 2015). We observed that decreased gene relative abundances for the type III secretion system and the QseBC two-component system were mainly assigned to *Escherichia*, which exerts a barrier effect against enteropathogens (Hudault et al., 2001), and are responsible for providing vitamin K2 (Meganathan, 2001), suggesting a depleted beneficial metabolism from *Escherichia* in diarrheic piglets. Other two-component regulatory systems, such as KdpDE, LiaS-LiaR and GlnK-GlnL, may involves in the utilization of glutamine that maintain homeostasis of cellular energy and stress response (Satomura et al., 2005), and thus play a crucial role in the development in diarrhea.

However, the metagenomic information available in this study represents only one step in the functional investigation of diarrhea-associated microbiota. Due to the small sample size of animals used in this study and the inter-individual variations in microbial composition and function, we failed to find the difference in relative abundance of the phylotypes at species level from our metagenomic data. Although there is evidence suggesting that *Prevotella*-correlated microbial aberration and dysfunction underpin the pathogenesis of piglet diarrhea, this striking finding requires to be validated in larger studies, for instances, those integrating the pathology and metabolism characteristics across a greater number of samples.

In summary, the colonization of *Prevotella* and *Fusobacteriaceae*, the concurrent decreases in *E. coli* and that the majority of beneficial bacteria belong to the *Firmicutes* phylum, underscores the crucial role of the microbiota in the development of neonatal piglet diarrhea. Major functional gene sets associated with piglet diarrhea are involved in: (i) increased potential to promote the overgrowth of bacteria using ribosome biogenesis; (ii) reduced potential to assimilate and utilize multiple energy sources and circumvent microbial infections using the transport systems of polyamine, monosaccharide, amino acid, and sugar-specific PTS, as well as two-component regulatory systems. Identification of these biomarkers may enable interventions to reduce the occurrence of neonatal piglet diarrhea.

## Author Contributions

SG conceived the experiment and revised the manuscript. QY performed the experiments, analyzed the data and wrote the manuscript. SgZ, SwZ, XH, PW, SL, and WH conceived and designed the study and edited the manuscript. LL, WS, and ZY participated in the analysis and interpretation of data.

## Conflict of Interest Statement

The authors declare that the research was conducted in the absence of any commercial or financial relationships that could be construed as a potential conflict of interest.

## Footnotes

1 http://drive5.com/uchime/uchime_download.html

## Supplementary Material

**FIGURE S1 | Taxonomic profiles of the fecal microbiome in diarrheioc and healthy piglets from metagenomic sequencing.** The relative abundance of the top 10 phyla **(A)** and the top 10 genera **(B)** of fecal bacteria present in both diarrheic and healthy piglets. Comparison of the fecal microbial profiles from diarrheioc and healthy piglets between the16S rRNA gene sequencing and the metagenomic sequencing data at the phylum **(C)** and the genus **(D)** level. MD and MH represents the diarrheic sample and healthy sample, M_D and M_H represents the metagenomic data from the diarrheic and healthy sample, 16S_H and 16S_D represents the16S rRNA gene sequencing data from diarrheic and healthy sample.

**FIGURE S2 | The distribution of KEGG functional categories in diarrheic and healthy piglets.** The bar chart displays a comparison of gene abundance summarized into KEGG functional categories to illustrate functional enrichments.

**FIGURE S3 | Principal component analysis (PCA) of the metagenomic samples using the KOs profiles.** PCA plots showed that functional profiles of the fecal microbiome in diarrheic piglets significantly differed from those in healthy subjects, and the variability in diarrheic piglet is higher than that in healthy subjects.
