# Supplementary figures and images for: Structure and Function of the Fecal Microbiota in Diarrheic Neonatal Piglets
Source: Front Microbiol. 2017 Mar 24;8:502. doi: 10.3389/fmicb.2017.00502 (PMC5364137; doi:10.3389/fmicb.2017.00502)

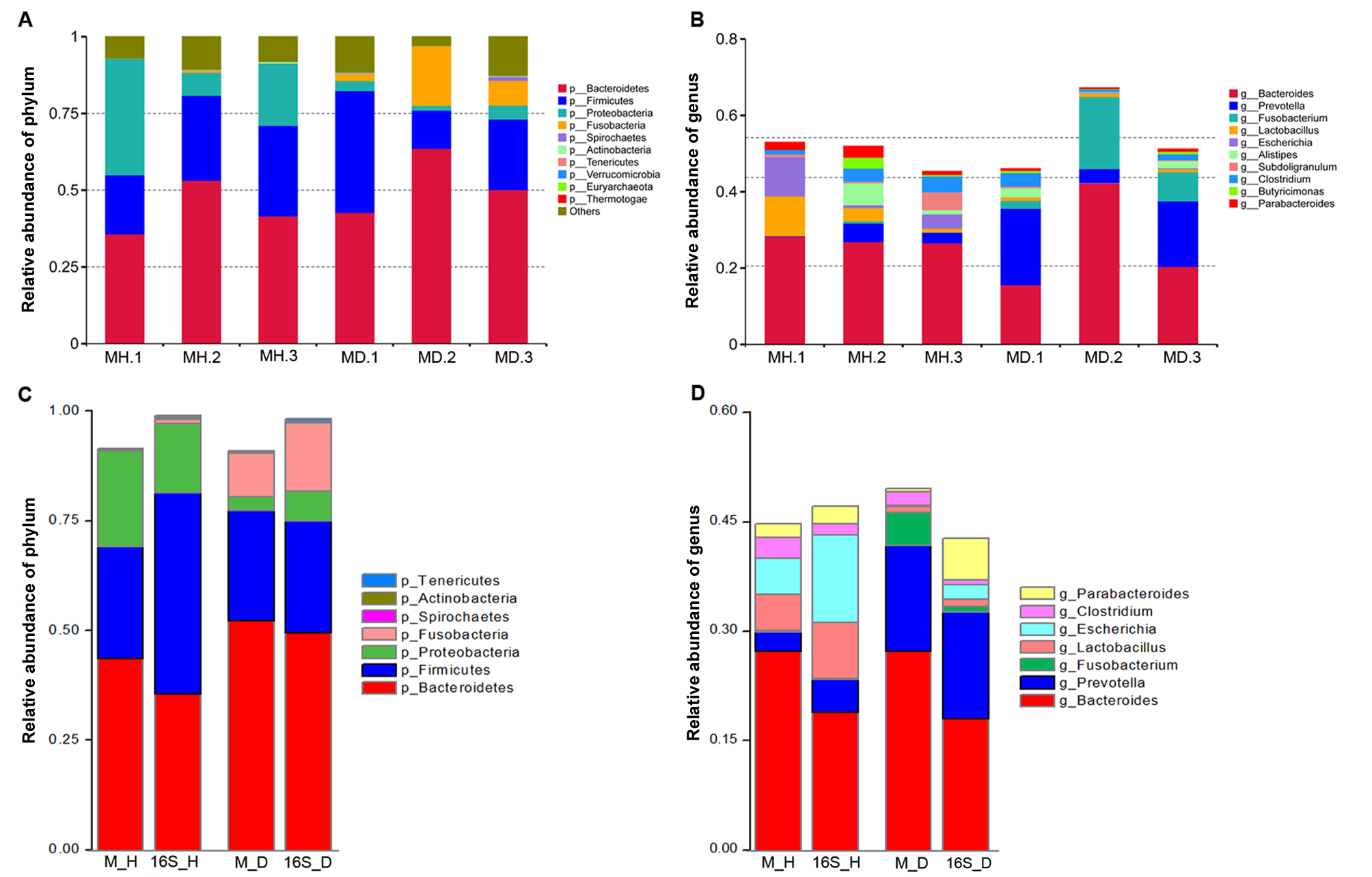

Supplement: Supplementary file 1 [file Image_1.TIF]

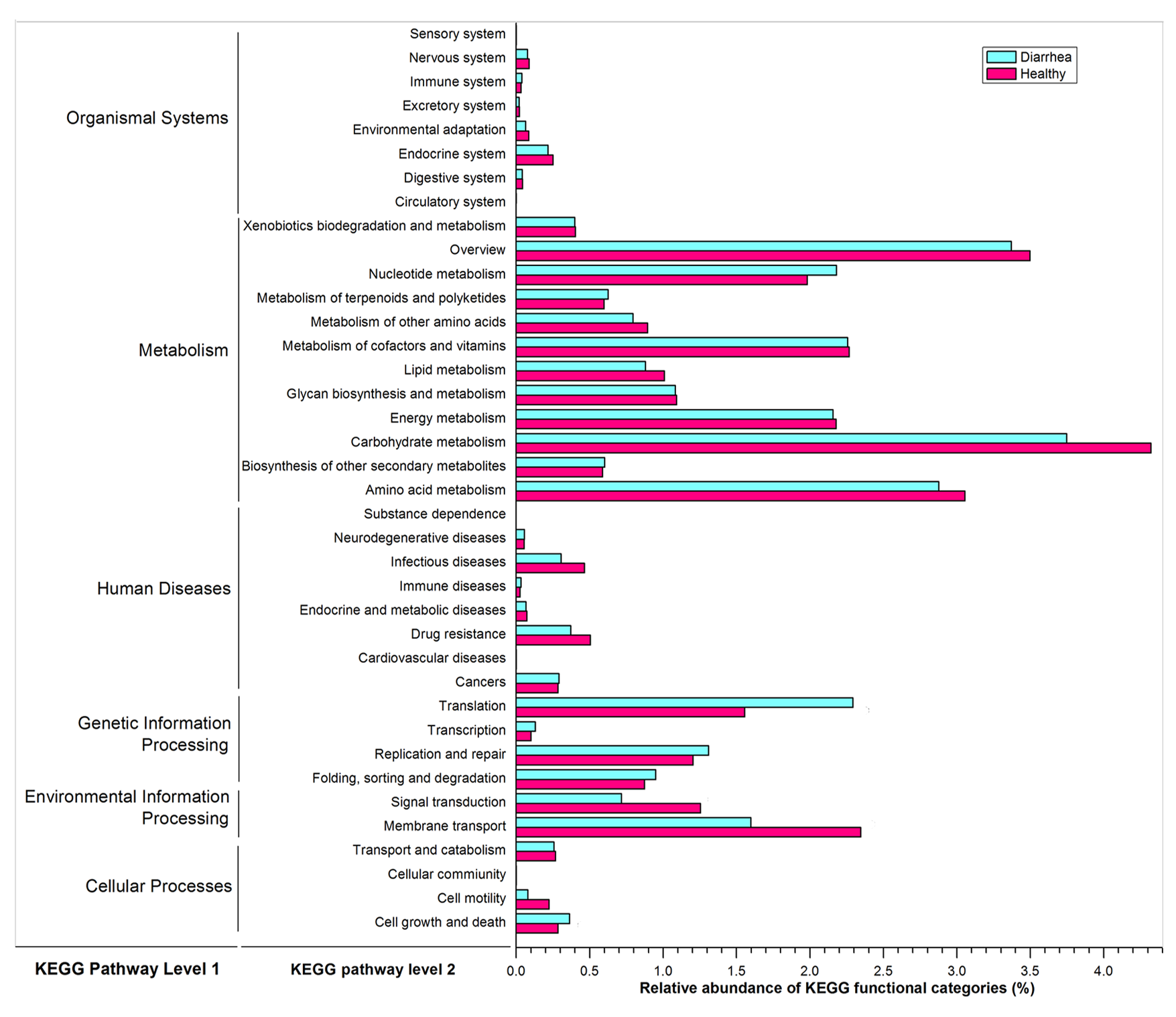

Supplement: Supplementary file 2 [file Image_2.TIF]

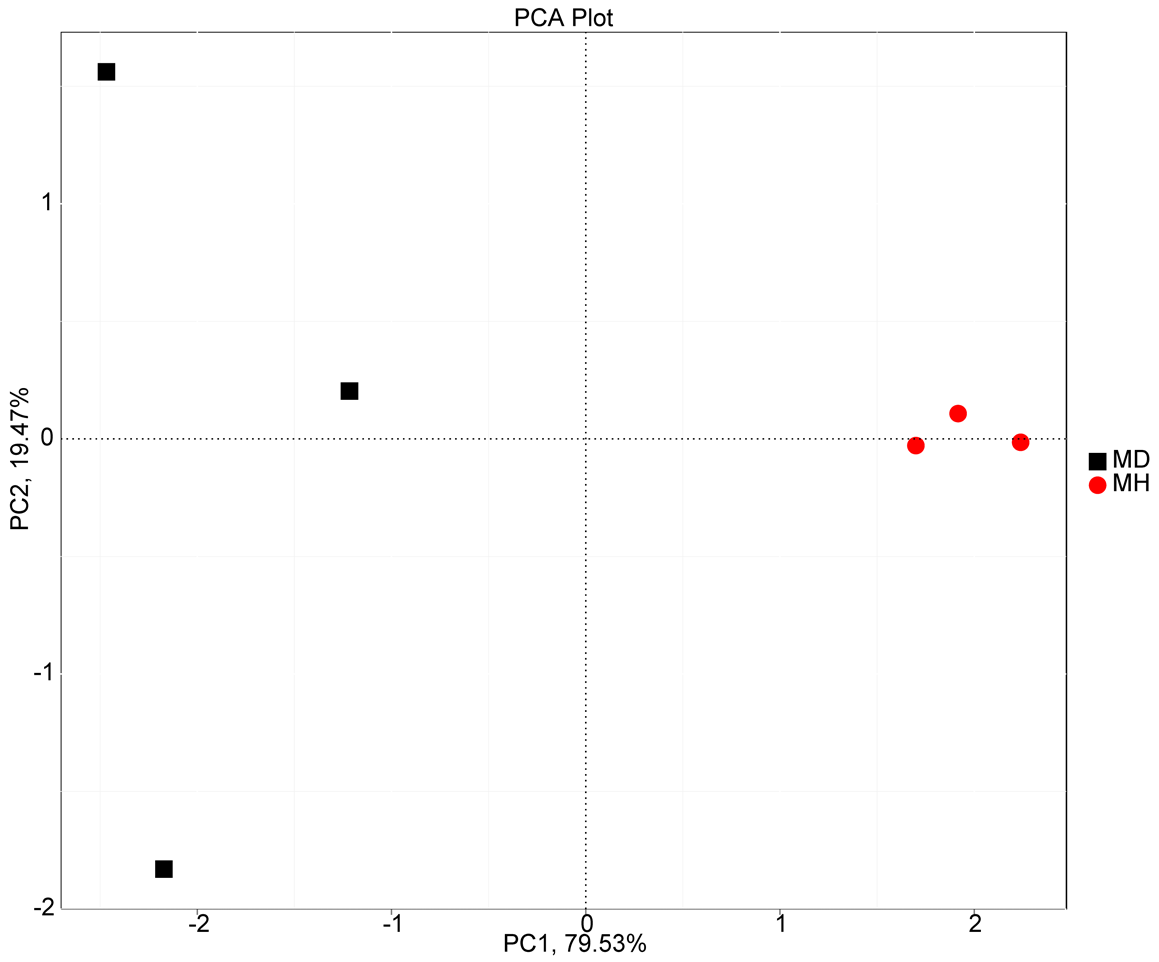

Supplement: Supplementary file 3 [file Image_3.TIF]
